# Isolation over 35 years in a heated biotest basin causes selection on MHC class IIß genes in the European perch (*Perca fluviatilis* L.)

**DOI:** 10.1002/ece3.1426

**Published:** 2015-03-05

**Authors:** Mats Björklund, Teija Aho, Jasminca Behrmann-Godel

**Affiliations:** 1 Department of Animal Ecology, Evolutionary Biology Centre, Uppsala UniversityUppsala, Sweden; 2 Department of Aquatic Resources, Institute of Coastal Research, Swedish University of Agricultural SciencesSkolgatan 6, Öregrund, SE-742 42, Sweden; 3 Limnological Institute, University of KonstanzMainaustrasse 252, D-78464, Konstanz, Germany; Guldhaven Pelagiska ABBox 43, SE-952 21, Kalix, Sweden

**Keywords:** MHC II, microsatellites, *Perca fluviatilis*, selection, time series, warming

## Abstract

Genes that play key roles in host immunity such as the major histocompatibility complex (MHC) in vertebrates are expected to be major targets of selection. It is well known that environmental conditions can have an effect on host–parasite interactions and may thus influence the selection on MHC. We analyzed MHC class IIß variability over 35 years in a population of perch (*Perca fluviatilis*) from the Baltic Sea that was split into two populations separated from each other. One population was subjected to heating from cooling water of a nuclear power plant and was isolated from the surrounding environment in an artificial lake, while the other population was not subjected to any change in water temperature (control). The isolated population experienced a change of the allelic composition and a decrease in allelic richness of MHC genes compared to the control population. The two most common MHC alleles showed cyclic patterns indicating ongoing parasite–host coevolution in both populations, but the alleles that showed a cyclic behavior differed between the two populations. No such patterns were observed at alleles from nine microsatellite loci, and no genetic differentiation was found between populations. We found no indications for a genetic bottleneck in the isolated population during the 35 years. Additionally, differences in parasitism of the current perch populations suggest that a change of the parasite communities has occurred over the isolation period, although the evidence in form of in-depth knowledge of the change of the parasite community over time is lacking. Our results are consistent with the hypothesis of a selective sweep imposed by a change in the parasite community.

## Introduction

Understanding the mechanisms that alter genetic diversity of functionally important traits between populations is a major focus of modern evolutionary biology (e.g., Rundle and Nosil 2005; Schluter 2009; Maan and Seehausen 2011). Functionally important traits are major targets of selection and studies of the diversity of their genes have found fundamental differences between populations (e.g., Futuyama 2013). One good studied group of these functionally important traits is the genes of the major histocompatibility complex (MHC). MHC genes belong to the most polymorphic gene families in the jawed vertebrates were the MHC constitutes an important component of the immune system shaping the immune response of an individual (Klein 1986). The MHC genes code for receptors that bind antigens derived from pathogens and present those to cells of the immune system, inducing the adaptive immune response. There are two classes of MHC genes: MHC class I receptors that present antigens stemming mainly from intracellular pathogens such as viruses and cancer infected cells and class II receptors that present antigens derived from extracellular pathogens such as parasites (Janeway et al. 2005; Sommer 2005). Thus, the specific MHC class II allele setting of an individual determines directly its capability to resist or defend specific parasitic infections. The extraordinary high genetic diversity of the MHC genes is believed to be maintained by a number of non-neutral mechanisms (Bernatchez and Landry 2003; Piertney and Oliver 2006). Three major mechanisms have been hypothesized, based on heterozygote advantage, negative frequency-dependent selection, and fluctuating selection (Spurgin and Richardson 2010). The heterozygote advantage hypothesis proposes that individuals heterozygous at MHC loci are better protected against parasitic invaders than homozygous individuals because they possess more receptors for the detection of pathogens and the possibility of triggering the adaptive immune response. This ability increases the fitness of heterozygous individuals in the population and thus maintains the diversity of MHC alleles (Doherty and Zinkernagel 1975; Hughes and Yeager 1998; for an example see Oliver et al. 2009b). The negative frequency-dependent selection hypothesis proposes that individuals carrying resistance alleles that are rare (or new) in the host population have a lower risk of being parasitized. This hypothesis is also known as the rare allele advantage hypothesis. Under this hypothesis, the coevolutionary processes between hosts and pathogens can lead to a cycling in the frequency of MHC alleles in the host population (Clarke and Kirby 1966; for an example see Paterson et al. 1998). The fluctuating selection hypothesis proposes that the allele diversity at the MHC is maintained by a fluctuation of parasites and pathogens in space and time such that individuals will face variable pathogens in different areas of the environment or during different seasons of the year. These environmental fluctuations may also lead to local adaptation and differentiation between subpopulations or demes (Hill 1991; for an example see Westerdahl et al. 2004). Whereas the process of balancing selection is the basis for the first two hypotheses outlined above, directional selection accounts for the fluctuating selection hypothesis (Spurgin and Richardson 2010). Additional mechanisms for the maintenance of genetic variability such as balance between selection at different levels (Maynard Smith 2001) may also be important for the maintenance of MHC variability. However, the exact determination which of these mechanism accounts for the maintenance of genetic polymorphism in a population is not easy to determine. All of these mechanisms are not mutually exclusive and may act in concert with other neutral and selective forces as well as with one another to maintain MHC allele diversity in various species (Apanius et al. 1997; Spurgin and Richardson 2010).

In addition, it has been shown that MHC genes can shape the individual body odor and can thus be the target of sexual selection via odor-based mate choice (Reusch et al. 2001; Penn 2002). Thus, by its pleiotropy, MHC genes have been hypothesized as being “magic traits”, accelerating and stabilizing divergent selection between populations (Eizaguirre et al. 2009b).

It is well known that the interaction between hosts and parasites is affected by the physical environment such as the temperature regime (Wolinska and King 2009). This may be especially true for poikilothermic organisms like fish. For example, the period of parasite transmission can be prolonged, the abundance of parasites can be increased, and the parasite community can be changed by a rising temperature (Marcogliese 2001; Hakalahti et al. 2006; Poulin 2006). In an experimental test, Landis et al. (2012) showed that an increase in water temperature decreased the proportion of innate immune cells in infected host pipefish (*Syngnathus typhle*). The results of this experiment indicates that there is a strong host × parasite × environment interaction that may affect the genes that play major roles in parasite defense such as MHC genes. Dionne et al. (2007) found a latitudinal gradient of water temperature and MHC class II allele variability and bacterial diversity in Atlantic salmon (*Salmo salar*) indicating selection on MHC class II variability with increasing parasite diversity and water temperature. During a natural heat wave in 2003 killing the majority of their experimental fish, Wegner et al. (2008) found a strong correlation between survival and the individual parasite load and MHC allele composition of sticklebacks. They showed that survival during increased water temperature was generally higher in families with an optimal (intermediate) number of MHC alleles and a lower parasite load.

**Figure 1:**
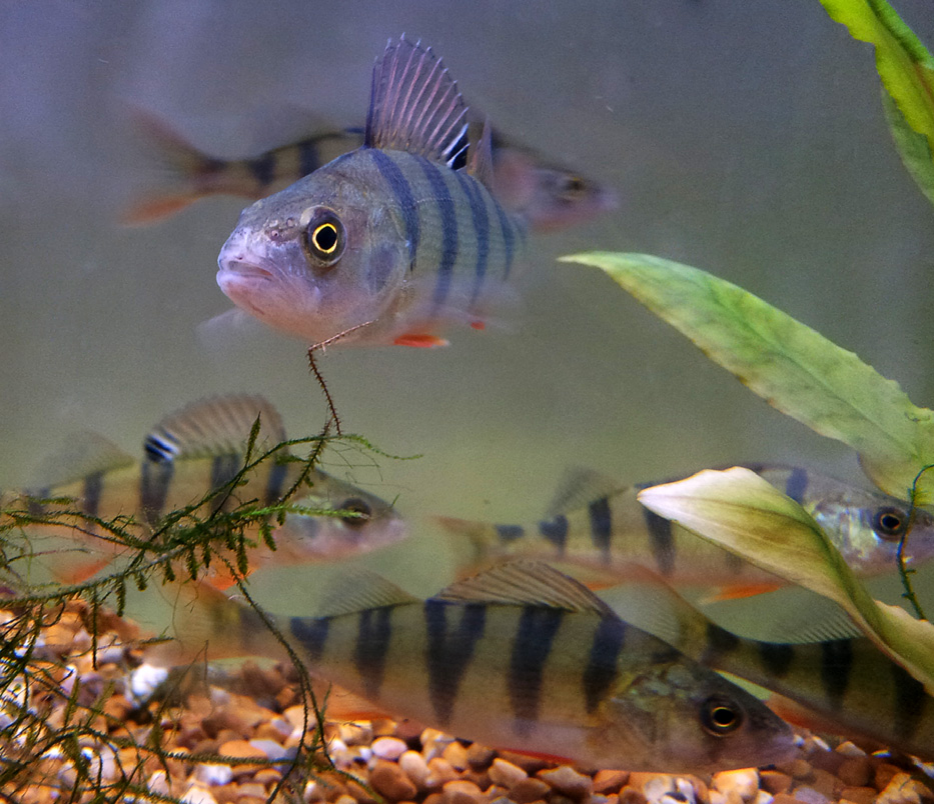
The study organism, the European perch *Perca fluviatilis*, is one of the most common freshwater fish in Europe. Picture taken by Fredrik Sundström, Department Animal Ecology, Uppsala University.

The European perch *Perca fluviatilis* (Fig.1) is an opportunistic widely distributed European fish species inhabiting rivers, streams, lakes, and brackish waters like the Baltic Sea (Kottelat and Freyhof 2007). The perch is a long-lived species (maximum recorded age of 21 years, usually to about 6 years (Kottelat and Freyhof 2007), with a generation time of approximately 3 years, reproducing once a year in spring (Craig 2000). Due to its broad distribution, the wide range of habitats used and the many food sources utilized, a high number of parasite species from various orders have been described for the species (e.g., Craig 2000; Morozinska-Gogol 2008; Behrmann-Godel 2013). In previous studies Michel et al. (2009) and Oppelt and Behrmann-Godel (2012) found a high variability of MHC class II ß genes in the perch with at least five expressed loci, and numerous alleles and established a genotyping method via reference strand conformation analysis (RSCA) which allows for high-throughput individual MHC genotyping (Lenz et al. 2009).

**Figure 2:**
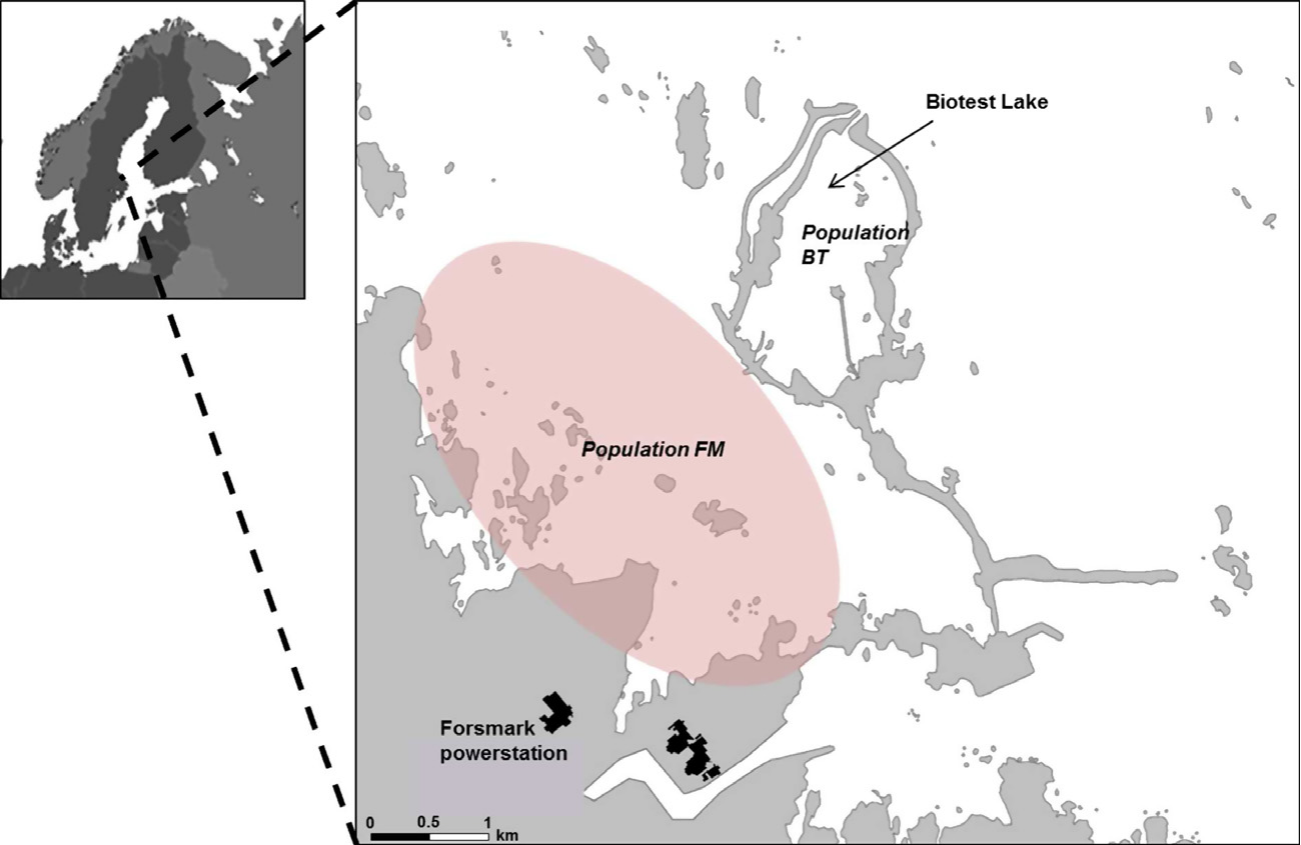
Map over the study area with the Biotest Lake and the control area (Forsmark).

In this study, we study how isolation and environmental change (heating) over a long period of time affect the MHC class II ß allele variability of European perch from the Baltic Sea in Sweden. By applying the predictions from different selective models for MHC evolution, we will enhance our understanding of the selective mechanisms provoking the observed allele frequency changes over time. We took advantage of a large-scale experiment where the cooling water from a nuclear power plant (Forsmark, Eastern Sweden) has been released into an artificial lake, the Biotest Lake, which was closed from the surrounding Baltic Sea to prevent fish migration from the start of 1977 to 2004 (Fig.2). During the 24 years, the population of fish in the Biotest Lake was isolated and the fish experienced a mean water temperature between 6 and 10 degrees higher all year round compared to the surrounding sea (Sandström et al. 1995; Karås et al. 2010). This is a major environmental impact especially in the summer period when water temperatures can be close to the upper physiological limit of fish adapted to temperate conditions (e.g., Beitinger et al. 2000). Likewise, one of the major intermediate hosts, *Radix balthica*, is known to suffer from higher temperatures (Cordellier and Pfenniger 2009; Verbrugge et al. 2012) and is now almost extinct in the Biotest Lake (Karås et al. 2010). Thus, the environmental conditions for parasites and hosts including their coevolutionary interactions (G × G × E) may differ considerably inside and outside the Biotest Lake. It has been shown that this long-term increase in temperature has resulted in a number of changes in the Biotest Lake perch population such as increased growth rate, larger absolute size, and changes in various life history parameters (Sandström et al. 1995, 1997; Lukšiene et al. 2000). The parasite community of perch has unfortunately not been monitored in detail over the years of enclosure. However, there are indications for a shift in the host community for certain fish parasites in the warmer Biotest Lake. For example, *Radix balthica* (synonym *Radix peregra, Lymnea ovata*) which is the most common gastropod in the Baltic Sea harboring a number of fish parasites including trematodes that are infective for perch (Niewiadomska & Kiseliene 1994; Behrmann-Godel 2013) has been replaced by the exotic New Zealand mud snail *Potamopyrgus antipodarum,* which is the gastropod with the highest densities in the BL at the present (Karås et al. 2010), but is not known as an intermediate host for the same parasites as *R. balthica* (Morley 2008; Karatayev et al. 2012). Thus, if the planktonic or snail community has changed due to the rise in temperature, the fish parasite community may have changed concomitantly.

**Figure 3:**
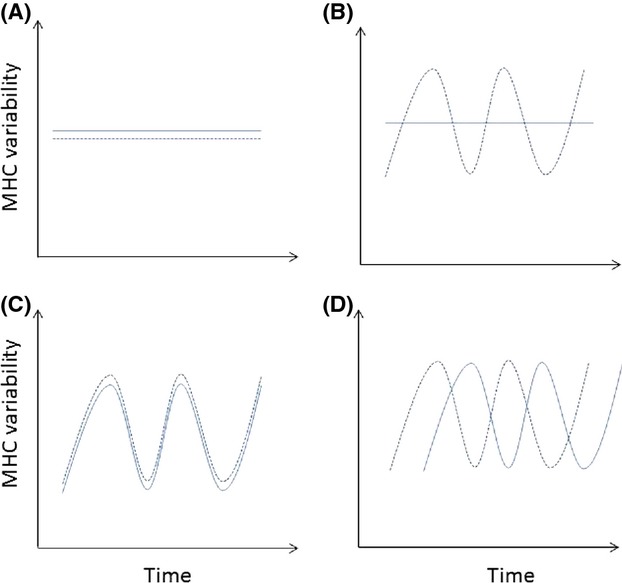
Summary of the different possible outcomes with regard to MHC variability over time in the two populations. (A) No change in either population, (B) change in the heated population, but not in the control population, (C) a scenario where the temporal change in both populations is the same, (D) a scenario where the temporal change in both populations is different.

Tissue samples of perch from Biotest Lake and a reference site outside at Forsmark (Fig.2) have been stored yearly, giving us the unique opportunity to study the long-term change in immune genes, MHC class IIß genes, as a direct indicator of a changing selection regime by parasites and pathogens. As the population and the physical environment was in origin the same, before the construction of the barrier, the populations inside and outside the Biotest Lake share the same history. This means that any differences evolving over time will be due to the isolation itself rather than any intrinsic differences at the two sites. This could be due to a bottleneck at the time of isolation with subsequent genetic drift, or due to selection as a result of changing environmental conditions, or a combination of both. While comparing two populations that differ in some environmental parameter could give insights, adding a temporal aspect gives far more information because it allows us to see what changes has occurred in each of the populations over time. Given the complexity of the models concerning MHC evolution only very general predictions can be made. First, if selection imposed by parasites is constant over time and the hosts have evolved a certain level of resistance, we would not see a change in MHC allele frequencies over time in any of the populations. Thus, allele frequencies in the two populations are stable and will not differ from each other at any point (Fig.3A). Second, if the isolation and the changed environmental conditions in the Biotest Lake have had an effect on the parasite community, we would expect to see MHC allele frequencies change in the Biotest Lake, but not in the outside control population (Fig.3B). If so, we could conclude that the isolation and the change in the environment have had an effect per se. Third, both populations may be driven by a constant arms race between parasites and hosts resulting in fluctuating selection on different MHC alleles over time. If these temporal changes are the same in the two populations, we can reject any influence of the isolation on the selection regime (Fig.3C). Fourth, if we see different patterns of allelic change in the two populations, we can reject the hypothesis that changes in the environment and isolation are not important and the selection regimes differ in both populations (Fig.3D). A crucial part is to disentangle the effect of isolation and change of environment as this happened simultaneously. If the isolation imposed a bottleneck, which can change allele frequencies substantially, we would see this at neutral markers such as microsatellites. If we can reject the existence of a bottleneck at the time of isolation, our inference that a changing environment is an important factor affecting the selection regime on MHC alleles. Hence, by comparing the two populations over time, we can reject some hypotheses even though a more detailed scrutiny of different hypotheses for the evolution of MHC variability might be difficult (cf Spurgin and Richardson 2010).

## Materials and Methods

### Sample area

The study area was the Biotest Lake outside the Forsmark nuclear power plant (Forsmark (east coast of Sweden, 60°25′N, 18°10′ E, Forsmark, Sweden; Fig.2). The size of the artificial lake is 90 ha with a mean depth of 2.5 m. This lake is artificial insofar as it was segregated from the surrounding sea where the control fish were sampled by connecting a series of natural islands with manmade dikes. Gratings were positioned in the north of the enclosure to allow water outflow, but to prevent fish migration between inside and outside the Biotest Lake. Thus, the control population experience physical conditions (air temperature, water depth etc.) that does not differ greatly from the Biotest Lake apart for the difference in water temperature. The water temperature is around 6–10 degrees warmer in the Biotest Lake than in the surrounding sea (Sandström et al. 1995; Karås et al. 2010). In winter, the Baltic Sea water temperature is around zero and regularly freezes while no freezing happens in the Biotest Lake due to the temperature being around 6–10 degrees. In the summer, the Baltic Sea typically has a temperature of around 20–25 degrees, while the Biotest Lake has a temperature range between 30 and 35 degrees. The gratings were removed in 2004, and the fish are since then able to swim freely between the Biotest Lake and the surrounding sea. However, in most of the year, the currents out from the Biotest Lake are strong enough to prevent a steady influx into the Biotest Lake from the outside area, while fish from the Biotest Lake can easily get out from the lake.

### Sample collection

The samples were taken from the Biotest Lake (BT) and from the control point Forsmark (FM) just outside the artificial lake between 1977 and 2009 (see Sandström et al. 1995 for details). Fish were collected several times yearly by the Institute of Coastal Fisheries using gill nets, but the fish used here were all from summer samplings. From the sampled fish, body length and weight was noted and operculae were removed, cleaned, dried, and stored at the Institute of Coastal Fisheries for later analyses. From the stored perch samples, 30 individuals were used for this study taken from every second year and population, with a total of 1020 individuals from 1977 to 2009 sampling. In order to sample perch from nonoverlapping generations that belonged to different cohorts, we used operculae from fish matched in body length (approximately around 30 cm TL). A small piece of the dried operculum was grinded and digested overnight in extraction buffer (as given in Aljanabi and Martinez 1997) containing proteinase K and 10% SDS. DNA was then extracted using the standard salt extraction procedure (modified after Aljanabi and Martinez 1997 and Paxton et al. 1996). Hence, we have a time series that contains 17 data points over 34 years for both populations.

### Genetic analyses

We analyzed nine microsatellite loci in two multiplexes using a Type-it kit (Qiagen, Limburg, The Netherlands). The first batch contained the markers Pfla2, Svi17, Pfla5, SviL7, Svi6, and PflaL10 and the second batch contained the markers Svi18, PflaL4, and Plfa9 (Borer et al. 1999; Wirth et al. 1999; Leclerc et al. 2000). The PCR cycle was 95° for 5 min, then 27 rounds using a cycle of 95° for 30 sec, 56° for 90 sec, and 72° for 30 sec, and we ended the PCR with 60° for 30 min. The two batches were fragment analyzed on an ABI 3730XL genetic analyzer. Allele calling was carried out automatically with the GeneMapper v4.1 (Applied Biosystems, Waltham, MA, USA.) software and controlled visually.

Sequences of the MHC class II ß1 domain (called “MHC alleles” hereafter) of individual perch were amplified using the primers pfluco1 (Oppelt and Behrmann-Godel 2012) and StviMH5R (Michel et al. 2009). The use of this primer set restricts the amplification to a fragment of the MHC class II exon II region restricted to approximately 200 bp of the ß1 domain, coding for the antigen binding site. It allows for amplification of expressed MHC alleles from at least five class II loci and omits expression of nonfunctional alleles (See Michel et al. (2009) and Oppelt and Behrmann-Godel (2012) for further details). However, single MHC alleles could not be assigned to distinct loci. Perch were genotyped by RSCA (reference strand-mediated conformational analysis) using two fluorescent reference strands, pefuDXB01 and pefuDXB08 (accession numbers: FB293126 and FN293147) following the protocol in Oppelt and Behrmann-Godel (2012).

A saturation curve showing the expected number of MHC alleles based on sample size was calculated and is given in Fig. S1 (Supporting Material). This was performed by resampling individuals from the combined data for all years at different sample sizes (10, 20… 400) and a counting of the number of alleles found in these new samples. This was repeated 10,000 times to obtain the 95% interval.

### Testing for genetic variation

We analyzed possible deviations from Hardy–Weinberg equilibrium by comparing the number of expected and observed heterozygotes in the microsatellite data. Fixation indices *F*_ST_ and *F*_IS_, test of linkage disequilibrium, Garza–Williamson M, and heterozygosity were calculated using the softwares Arlequin (Excoffier and Lischer 2010), FSTAT (Goudet 2001) and Genepop (Raymond and Rousset 1997). 95% intervals for the pairwise values of *F*_ST_ from the microsatellite data were obtained from the randomization procedure in Arlequin.

To calculate pairwise *F*_ST_ values from the MHC data, we used the relative frequencies of the alleles observed in each population and calculated *F*_ST_ using the standard way (e.g., Hartl and Clark 1989, pp 293–4). We created 95% intervals (adjusted for the number of tests using Sidak's correction, (true α = 1 − (1 − α)^1/*t*^, where t is the number of years) by randomizing the alleles (rather than individuals as was performed for the microsatellites) between the two populations compared. This was repeated 10,000 times. Using this standard way of *F*_ST_ calculation, we were aware that the results might be biased for the following reason. Based on the use of the general primer set which amplifies alleles from several loci and our specific genotyping procedure (RSCA), we were not able to assess the true number of alleles per individual. This is simply based on the fact that alleles in homozygote states cannot be differentiated from alleles in heterozygote states and are thus regarded as one allele rather than two identical alleles. This will bias the calculation of allele frequencies and thus also bias the calculation of pairwise *F*_ST_ values. To evaluate the magnitude of this bias and to find out whether we would have to correct for it, we used a simulation approach (details in Data S1 supporting material). From this simulation, it is obvious that the *F*_ST_ values using the observed allele frequencies without correction for “zygosity status” of the loci were overestimated to some extent. However, as is obvious the magnitude of the bias is small in relation to the sampling error and hence we refrained from further correcting for this bias in the calculations of the pairwise *F*_ST_ values.

We calculated allelic richness (AR), which is known to be strongly affected by reductions in effective population size (Leberg 2002) and has been shown to be the most sensitive measure of a reduction in genetic variability (Hoban et al. 2014). AR was estimated in FSTAT (for the microsatellite data only) and was calculated as the number of alleles taking sample size into account using a rarefaction approach. For MHC, we calculated the total number of alleles in each year as an estimate of allelic richness. The number of alleles was calculated assuming the maximum number of loci (see above and Data S1), summed over all individuals in a particular population and year. This means that the calculated number of alleles will be higher than the observed number of alleles to some unknown extent depending on the degree of heterozygosity of the MHC loci. As we are not sure how many gene copies we have in each individual, we used a slightly different rarefaction approach. We created an expected distribution of AR over time assuming that all differences between years in terms of AR are due to sampling alone and thus that the frequencies have not changed over time. This was accomplished by repeated multinomial sampling (10,000 times) using the different sample sizes (number of alleles) from the different years using the total dataset as a reference. This gives an expected total number of alleles (calculated as a maximum see above and Table S2) given the sample size and the allele frequencies and a 95% confidence interval adjusted for the number of tests using the Sidak's correction. In addition, we calculated gene diversity (Nei 1987) and estimated 95% intervals in the same way.

### Testing for population bottleneck

We estimated effective population size, *N*_e_, using the software LDNe (Waples and Do 2008) and for the temporal method in NeEstimator (Do et al. 2014) for the microsatellite data only. We only used alleles with a frequency of at least 0.05 in the analysis because allele frequencies close to 0 or 1 can affect the estimation in an unknown way (Waples 2006). We estimated the confidence intervals by jackknifing (included in the program LDNe). The highest values were set to 2000 because the program returned negative values indicating infinite populations (Waples and Do 2008); hence, the estimates of *N*_e_ might be biased downward.

### Testing for temporal patterns in the time series

We used the following approach to analyze temporal patterns in allele frequencies. First, we tested for autocorrelations over the whole time period using the Box–Ljung *Q*-test (Ljung and Box 1978). A significant test up to maximum lag (*N* − 4 = 13) shows that the time series as a whole deviate from white noise, that is, is nonrandom. However, the time series could also deviate up to a certain point, which can be indicated from the test. Second, we calculated cross-correlations between the MHC alleles in each population and between corresponding alleles in the two populations. It is well known that autocorrelations inflate the cross-correlations and thus we removed these by differencing (*Y*(*i*) − *Y*(*i* − 1); Chatfield 2004). Third, we smoothed the periodogram by means of Parzen smoothing with a window size of 8 (=2√*N*, where *N* = number years, from Chatfield 2004; pp 134–136). After smoothing, we analyzed the periodograms by means of Fourier spectral analysis to find the period of cycles in the time series if the white noise null hypothesis was rejected (for details see Chatfield pp 121–146). We also fitted the behavior of two of the alleles (see Results) to the model of Decaestecker et al. (2013: eq 1) as a heuristic to visualize the cyclic behavior.

## Results

### Genetic diversity of MHC genes

**Figure 4:**
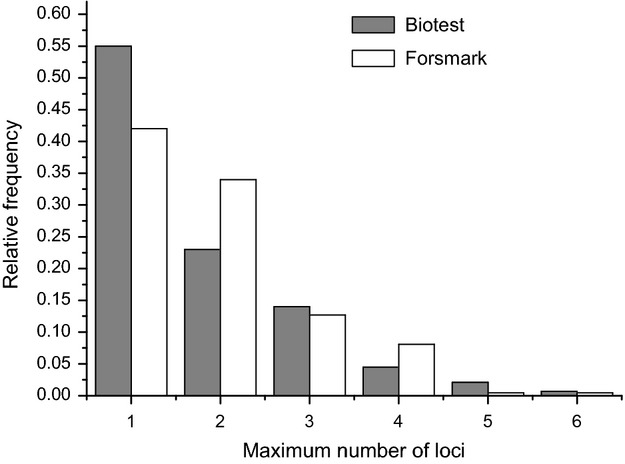
Relative frequency of the maximum number of MHC loci summed over all individuals and years in the Biotest Lake and Forsmark.

Amplification success of MHC alleles in the different years ranged between 33 and 90% in BT and between 37 and 97% in FM. Sample sizes for each year and locus are given in Table S2 (supporting material). In total, we found 51 different MHC class II alleles in all perch sampled during the whole time period. However, whereas all alleles (51) were present in FM, only 41 were found in BT resulting in 10 private alleles in the FM versus none in BT (see Table S2 Supporting Material). Interestingly, during the time of isolation (years 1979–2003), the mean number of different MHC alleles was nine in BT versus 16 in FM. The mean number of MHC alleles per individual did, however, not differ between the populations, albeit being very close to a significantly reduced number of alleles in BT (Biotest mean = 1.74, SD = 0.58, FM mean = 1.92, SD = 0.45, *Z* = 1.87, *P* = 0.061, Wilcoxon test; Data S1). The change in the individual number of MHC alleles over the years within BT was significant (Table S2). It changed from two alleles per individual to only one allele over a long time period between 1987 and 2001 before it rose to two alleles again (*Z* = −2.50, *P* = 0.011, Runs test). In FM, no such pattern was seen (*Z* = −0.28, *P* = 0.77, Runs test). We estimated the maximum number of loci in the two populations and the frequency distribution of number of loci combined overall years and individuals differed significantly between populations (*P* << 0.001; Fig.4). The maximum number of loci ranged from one to six (Table S2).

### Genetic diversity of microsatellites

For the microsatellite analysis of BT, 78–88% of all samples could be successfully amplified across the nine loci, and the corresponding figures for FM were 86–96%, even though in the 1981 samples three loci did not amplify at all (Pfla2, SviL7, and PflaL10).

In total, we found 221 microsatellite alleles over all loci in BT (mean = 24.6 and SD = 10.9), and 217 in FM (mean = 24.1 and SD = 11.1; Data S1). This difference was not significant (*P* = 0.73, Wilcoxon test). We found in total 25 unique alleles in BT (mean = 2.8 and SD = 2.0) and 24 unique alleles in FM (mean = 2.7 and SD = 1.8), and this difference was not significant (*P* = 0.83, Wilcoxon test).

We found three deviations (3/(17 years × 9 loci) = 3/153 = 2%) from Hardy–Weinberg proportions in BT, and none in FM across all years and loci. We found nine significant cases of linkage disequilibrium across all loci and years in BT (9/153 = 5.9%), and seven cases in FM (7/135 = 5.2%). This is very close to the expected number of significant tests by chance (=5%).

### Changes in genetic variability of MHC and microsatellites over time

**Figure 5:**
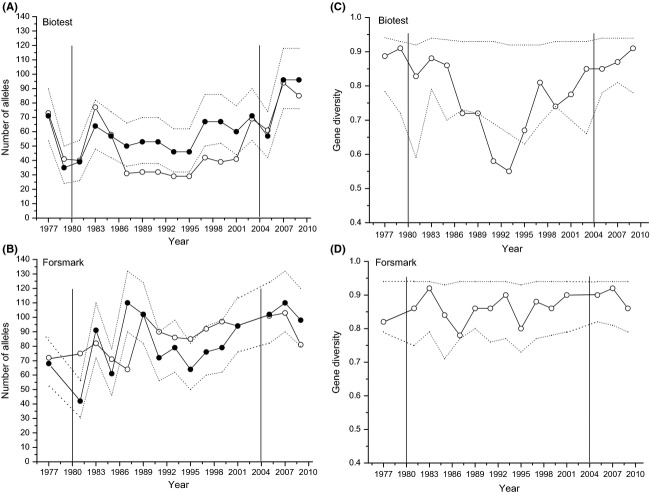
Allelic richness measured as the maximum number of alleles possible for (A) BT MHC and (B) FM MHC and MHC gene diversity for (C) BT, and (D) FM. The open dots are the observed values, the closed dots are the expected number based on rarefaction, and the dotted line represents the experiment wise 95% confidence interval. The vertical lines represent the year when heating started (left line) and the year when the barriers were open (right line). The time between these lines represent the time when the Biotest Lake was both heated and closed from migration.

Before the BT lake was closed from the surrounding sea, both populations had a comparable number of MHC alleles (BT = 18, FM = 14, *P* = 0.31, randomization test). Allelic richness, measured as the total maximum number of alleles (see Methods and Data S1) of MHC alleles, changed over the years in both populations. In BT, allelic richness was significantly lower than expected in eight consecutive years (Fig.5A), a pattern not seen in the FM population (Fig.5B), where the changes did not differ from what would be expected by sampling alone. At the end of the time period, however, after opening of the barriers in 2004, the allelic richness in BT strongly increased and thus in the end of the investigation period (2007 and 2009), both populations had again very similar allelic richness. The maximum number of MHC loci changed in a nonrandom way in BT (*Z* = −2.67, *P* = 0.0047, Runs test, Data S1), but randomly in FM (*Z* = 0, *P* = 1, Runs test, Data S1). The gene diversity of MHC dropped in BT between 1984 and 1993 and was significantly lower than expected between 1987 and 1993 (Fig.5C), while this was not observed in FM where gene diversity stayed high over the whole time period (Fig.5D).

The coefficient of variation (CV = standard deviation/mean) over the years of the most common allele of the different microsatellite loci did not differ between the two populations (BT: CV = 0.4 and FM: CV = 0.3, *P* > 0.76, Wilcoxon test); hence, they were combined in the following. This was not the case for the MHC alleles where the coefficient of variation was significantly larger in BT than in FM (BT: CV = 1.7 and FM: CV = 1.2, *P* = 0.028, Wilcoxon test). The microsatellite alleles varied significantly less than the MHC alleles in both populations (BT: *P* < 0.001 and FM: *P* < 0.001, Wilcoxon test).

**Figure 6:**
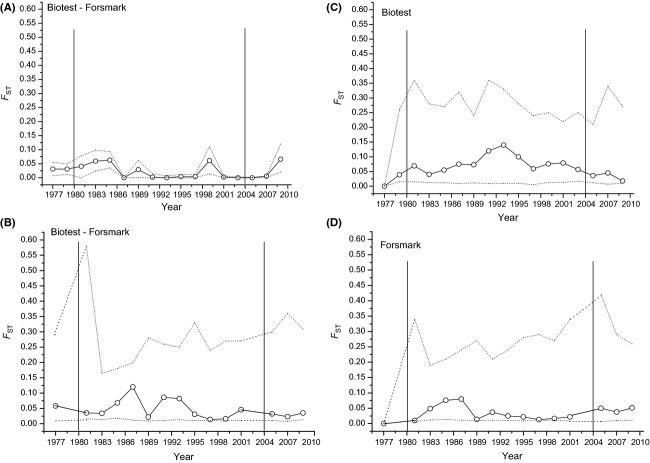
Pairwise *F*_ST_ comparisons over time. Given are the year-by-year comparisons between Biotest Lake and Forsmark perch populations based on (A) microsatellite data and (B) MHC data. The within population pairwise *F*_ST_ comparisons based on MHC data where every year is compared to the first year (1977) for Biotest (C) and Forsmark (D). The dotted line is the experiment wise 95% interval. The vertical lines represent the year when heating started (left line) and the year when the barriers were open (right line).

Pairwise *F*_ST_ comparisons for every sampling year between BT and FM based on microsatellite data were low and not significant overall years (Fig.6A). A similar result was found for pairwise *F*_ST_ comparisons based on MHC data. However, the *F*_ST_ values were generally much higher than the ones based on microsatellites (Fig.6B). In the comparisons between the first (1977) and all subsequent years, pairwise *F*_ST_ comparisons based on MHC data increased in BT between 1977 and 1993 to an *F*_ST_ value of 0.12 and decreased thereafter but stayed at levels of about 0.05 between 1997 and 2003. After opening of the barriers, *F*_ST_ decreased again to almost zero in 2009 (Fig.6C). In contrast, in FM, the pairwise *F*_ST_ comparisons stayed close to zero over the whole time period except a slight increase followed by a decrease between 1981 and 1989. After opening of the barriers, the *F*_ST_ values slightly increased (Fig.6C). However, due to the large confidence intervals in all *F*_ST_ comparisons based on MHC data, no significant differences in any of the comparisons were found.

**Figure 7:**
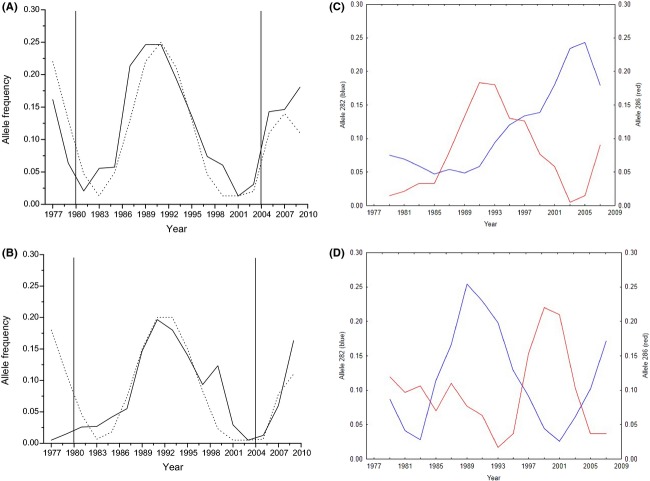
Change of allele frequencies in the two most common MHC alleles in both populations over time. The time series in (A) and (B) are smoothed with a Parzen smoothing window size 5, and the graphs (C–D) using a moving average (*N* − 3) procedure. (A) allele 282 in BT with the model (dotted line) of Decaestecker et al. (2013) fitted to the data (solid line), (B) allele 286 in FM fitted with the same model, (C) alleles 282 and 286 in FM, (D) alleles 282 and 286 in BT. The vertical lines in (A) and (B) represent the year when heating started (left line) and the year when the barriers were open (right line).

We found significant deviations from a white noise (“random”) model over the whole time period in the two most common MHC alleles in both populations (BT: allele 282: *Q* = 30.25, *P* = 0.0043, FM: allele 282: *Q* = 26.63, *P* = 0.014 and BT: allele 284: *Q* = 30.63 *P* = 0.0038, FM: allele 284: *Q* = 28.13, *P* = 0.0087), and in lags up to five time periods in allele 286 in BT (*Q* = 11.49, *P* = 0.043), and up to 10 lags in allele 286 in FM (*Q* = 18.91, *P* = 0.042). None of the microsatellite alleles showed this pattern (not shown). A cyclic pattern was found in the MHC allele 282 in BT with a period of 15 years according to five generations assuming a generation time of 3 years (Fig.7A). This was also found in allele 286 in FM with a period of 16 years (Fig.7B). The fluctuations of the BT allele 282 fit the theoretical model better than the FM allele 286 (Fig.7A, B). There were no significant cross-correlations between the MHC alleles in BT and those corresponding alleles in FM (282: *r* = −0.18; 284: *r* = 0.051; 286: *r* = 0.062; *P* > 0.2 in all cases). There was a significant negative correlation between alleles 282 and 286 in FM (*r* = −0.54, *P* = 0.046; Fig.7C), but not in the BT (*r* = −0.28, *P* = 0.28; Fig.7D).

### Test of possible bottleneck

**Figure 8:**
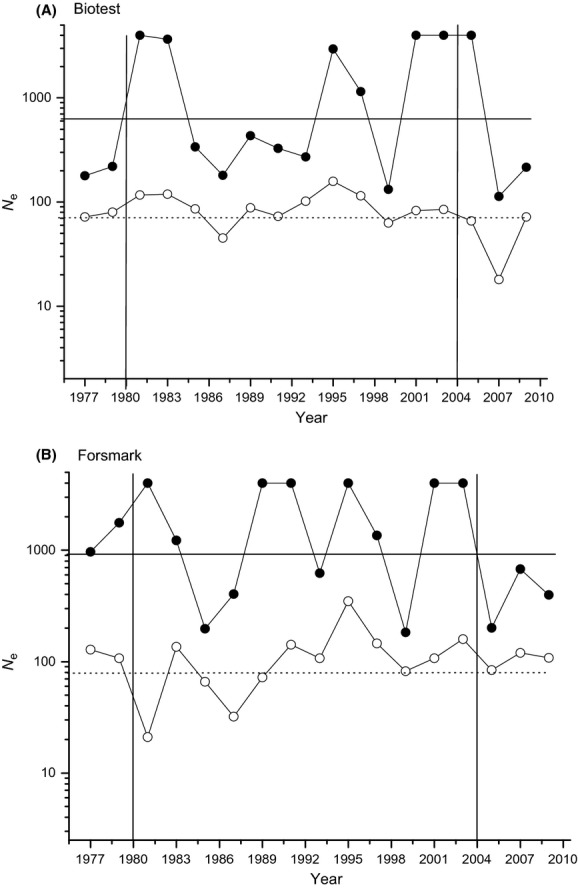
Effective population size (*N*_e_) in the (A) BT and (B) FM populations. Filled circles are the yearly estimates, and the open circles the yearly lower 2.5 percentiles. The solid horizontal line is the harmonic mean overall years, and the dotted line the harmonic mean of the lower 2.5 percentile overall years. The vertical lines represent the year when heating started (left line) and the year when the barriers were open (right line).

Effective population size was high in both populations over time. The harmonic mean of *N*_e_ over the years in BT was 537, and the lower 2.5% was 144 (Fig.8A). The corresponding figure for FM was 554 and the lower 2.5 being 89 (Fig.8B). In most years, the estimate of *N*_e_ was infinite and this was particularly true for the upper 95% figures. We set the maximum *N*_e_ to 2000, and the harmonic mean is calculated using this figure, and thus the estimates of *N*_e_ are conservative. Direct estimates of population size (*N*) from catch data that were estimated for the years 1982 and 1983 (O. Sandström pers. comm) suggest that the population of reproductive individuals in BT was around 20,000. This gives a *N*_e_/*N* ratio of 0.025, which is lower than in many other studies. If we assume a *N*_e_/*N* ratio of 0.1, we get an estimate of *N*_e_ around 2000. Using the temporal method in NeEstimator, we found that *N*_e_ in the BT was 451 (95% interval = 198–3356), and in FM, the program returned infinite, that is, a very large effective population sizes.

The allelic richness of microsatellites did not differ from the first year in the two populations at any year and the populations did not differ from each other (*P* > 0.1, Wilcoxon test) nor was there any pattern over time. The observed number of alleles did not differ from what could be expected given the allele frequencies and the sample size for any locus, year, or population (not shown).

## Discussion

### Selection on MHC alleles

By taking advantage of a long-term experiment of isolation and temperature rise in an enclosed population of European perch in the Baltic Sea, we could show a shift in selection regime on immune traits. The isolated Biotest Lake population of perch changed dramatically in allelic composition of MHC class II genes a short time after being isolated from the outside sea. The number of MHC alleles in the BT fish decreased during the time of isolation, a pattern that was not seen in perch from the control population. MHC allele frequencies varied much more in both locations compared to the microsatellite allele frequencies just as one would expect to see in loci under selection. This resembles results from sticklebacks where the variability in MHC alleles was higher than in microsatellites comparing various sympatric and allopatric stickleback pairs in Vancouver (Matthews et al. 2010). Similar results were also found in nine successive cohorts of the great reed warblers *Acrocephalus arundinaceus* where variability at MHC alleles exceeded the variability of microsatellites (Westerdahl et al. 2004).

The coefficients of variation of MHC alleles were significantly higher in BT compared to the control population (FM) indicating a higher rate of change in the MHC allelic composition in the BT perch population. Furthermore, we can rule out that the differences seen was a result of a bottleneck and subsequent genetic drift because the microsatellite loci analyzed did not show any pattern of change over time, and the estimates of effective population size was in general very high in both populations. Thus, the changes in MHC allele structure that we observed in the Biotest Lake perch, but not in the control population in Forsmark, can be attributed to a changed selection regime correlated to the enclosure of the fish community and the drastic long-term increase in water temperature. However, increased water temperature by itself will not have a selective effect on MHC alleles that could explain the observed patterns. Thus, the change seen in the MHC alleles in the BT perch population could be the result of a change in the selection regime by some temperature sensitive environmental factor. The isolation of a part of a continuous habitat is likely to induce a number of changes in the ecosystem as well as in the physical environment. This means that we cannot decisively conclude that the main factor causing the change in the selection regime is the drastic change in temperature. However, given what we know about the importance of ambient temperature in poikilotherm animals like fish and in invertebrates, the likelihood of the change in temperature being a main factor, directly or indirectly, shaping the selection regime and resulting in the observed change in MHC allele variability of perch must be considered to be very high.

We know that in the mid-1980s (shortly after heating started in Biotest lake), the intensity of the fish parasitic eye fluke *Diplostomum baeri* found in the eyes of perch was generally significantly higher in the Biotest Lake than in the outside sea (Höglund and Thulin 1990). In contrast, a survey in 2014 showed that fish from the surrounding sea (FM) had the same *D. baeri* intensities in their eyes as in the 1980s, but the intensity is now significantly lower in fish from the Biotest Lake ((Schmid 2014; unpublished Master′s thesis), indicating either a massive decrease of *D. baeri* in the Biotest Lake or that perch in Biotest Lake have developed immunological protection against *D. baeri* infections, or both. Additionally, the parasite *D. spathaceum*, which was common in the Biotest in the 1980s, was not found in 2014. Furthermore, a significantly higher frequency of perch with swollen kidneys was detected in Biotest Lake in 2004 as compared to fish from outside (50 and 25%, respectively) indicating a parasitic infection such as with myxosporidia (Alfjorden et al. 2003). The high prevalence of fish with swollen kidneys has not been recorded in any natural population in Sweden. This indicates that the parasite pressure could be higher, or at least different, in the warmer Biotest Lake than in the outside population. In 2014, 120 adult perch, 60 from FM and 60 from BT, were caught during June/July (Schmid 2014; unpublished Master′s thesis). Eyes, gut, and liver of all fish were analyzed for infections with macroparasites. All parasites were counted and determined to the species level. A community analysis in Primer 6 vers. 6.1.15 (Clarke 1993) revealed significant differences between perch from both locations (ANOSIM: *R* = 0.3, *P* < 0.01). Parasitic eye fluke infestations (*Diplostomum baeri* and *Tylodelphys clavata*) where significantly reduced in BT as compared to FM and contributed most to the differences in parasite community (SIMPER: *D. baeri* average dissimilarity = 13.2, contribution = 33%; *T. clavata* average dissimilarity = 9.2, contribution = 23%). This last analysis shows that despite barriers are open between BT and the surrounding sea since 2004 perch in both locations differ in their parasite communities. Thus, we have evidence that there have been changes in the parasite community over the years.

Similar results have between found between closely related limnetic and benthic stickleback species *Gasterosteus acculeatus* inhabiting different lakes in Vancouver. Limnetic and benthic sticklebacks from the same lake differed in terms of parasite communities (MacColl 2009), and limnetic sticklebacks had fewer MHC alleles per individual than benthic species (Matthews et al. 2010). Lake and river populations of sticklebacks from Northern Germany differed in both their parasite communities and MHC allele variability, and it was shown that river sticklebacks had less parasite species and fewer MHC alleles than lake sticklebacks (Scharsack et al. 2007, Eizaguirre et al. 2010). For these populations, a pleiotropic role of MHC genes was suggested to drive divergence between ecotypes due to parasite-mediated selection and assortative mating based on MHC-related mate choice (Eizaguirre et al. 2009a). These findings indicate that in sticklebacks, directional divergent selection on MHC genes driven by parasite-mediated selection followed by local adaptation might be intensified by the pleiotropic role of MHC during mate choice. However, Fraser and Neff (2010) found a contrasting role of MHC in Trinidad guppies where MHC variability was lower in comparison with neutral microsatellites comparing a number of wild guppy populations from various rivers in Trinidad (Fraser et al. 2010). The authors suggested stabilizing selection on MHC variability in these systems resulting in high similarity of MHC alleles between allopatric populations from the same river although they experienced differences in parasitism (Fraser and Neff 2010).

For the two most common alleles, the patterns of MHC allelic change in the Biotest Lake strongly suggest a temporal change in the selection regime in both populations. However, the pattern of MHC allelic change differed much between populations. The MHC allele 282 showed a clear cycling pattern in the BT population, whereas no such pattern was seen in the FM population (Fig.7A, C). The opposite was found for allele 286 that showed a clear cycling pattern in the FM population, whereas no cycling pattern could be found in the BT population (Fig.7B, D). The cycling pattern of allele 282 in BT (Fig.7A) fitted the theoretical model of Decaestecker et al. (2013) better than the cycling pattern of allele 286 in FM (Fig.7B). Thus, the pattern of change of all three alleles differed between BT and FM during the investigation period. A pairwise comparison of MHC allele frequencies between BT and FM showed no population differentiation and the allele frequencies of alleles 282, 284, and 286 were similar at the first sampling years in both populations (compare 1977 and 1981 in Table S2 (supporting material). This result indicates that the two populations did not differ in their MHC allele patterns before enclosure of the Biotest Lake. There are two possible explanations to the difference in cycling behavior of two of the alleles: (1) The change in environmental conditions had no influence on the cycling behavior of MHC alleles because differences in cycling patterns were already present before enclosure and stayed unchanged, or (2) the change in environmental conditions altered the cycling patterns of MHC alleles. As we did not find significant differences in initial allele frequencies between both populations and as they are derived from the same original population, the second explanation has more support from the data. Thus, we suggest that the change in environmental conditions resulted in a change of the cycling pattern of several MHC alleles in the Biotest Lake. However, the major difference in MHC allele patterns between both populations is the loss of allelic richness in BT during the time of isolation, which strongly suggests that selection has occurred (Fig.5A).

Thus, our initial equilibrium hypothesis of no change (Fig.3A) was clearly rejected, as was the hypothesis that selection occurred only in the BT population (Fig.3B) or that the two populations have experienced the same selection over time which would have led to a pattern of congruent change over time (Fig.3C). Even if the changes observed in the Biotest Lake are greater than in the control population, the control population has not been stable over time, as some of the alleles have varied significantly over time even in this population. This strongly suggests a scenario where selection occurred in both populations, but the selection regimes were different in both populations (Fig.3D). Thus, allele frequencies shift over time even in unmanipulated conditions. Whereas spatial variation of MHC alleles between populations or closely related species has already been shown for a number of species such as threespine sticklebacks (MacColl 2009; Eizaguirre and Lenz 2010; Matthews et al. 2010), water voles *Arvicola terrestris* (Oliver et al. 2009a), and house sparrows *Passer domesticus* (Loiseau et al. 2009), studies on temporal fluctuations of MHC allele frequencies over long time periods are rare. Our long-term time series analysis is thus one of the few existing datasets that empirically analyses MHC allele frequency changes over time and indicates the existence of fluctuating selection and specially frequency-dependent selection of MHC alleles in wild animal populations. Similar findings have been shown for great reed warblers (Westerdahl et al. 2004), Soay sheep (Charbonnel et al. 2005), and for non-MHC immune genes, (TAP) in brown trout *Salmo trutta* (Jensen et al. 2008). However, our analysis is the first to our knowledge indicating that environmental change, in this case most probably heating, may strongly impact the pattern of allele frequency change. Future analysis including more populations and also laboratory studies investigating the impact of temperature change on MHC × host × parasite interactions will expand our understanding of the selective processes that may drive population divergence and thus add to future biodiversity.

### What mechanism could have caused the observed changes in MHC between both populations?

A genetic change can be expected in a closed population if the isolation resulted in a bottleneck and subsequently low effective population sizes. If there was a bottleneck in BT, we would expect, for example, a reduced effective population size and a loss of allelic richness. In the microsatellite markers, we did not see any reduction in allelic richness over time, and the other tests of a potential bottleneck were also negative. Moreover, our estimate of the effective population size was high enough to make drift an unlikely explanation in BT.

Our results are consistent with a scenario where the parasite–host interactions have changed over time in the Biotest Lake. Due to warming, new intermediate host species such as exotic aquatic snails have successively established and obtained high population densities in the Biotest Lake replacing established species. As already mentioned, the snail *Radix balthica,* known to be an important intermediate host for several fish parasite species (Niewiadomska & Kiseliene 1994; Behrmann-Godel 2013), is almost extinct in BT and has been replaced by the exotic New Zealand mud snail *Potamopyrgus antipodarum* (Karås et al. 2010), but this species is not known as an intermediate host for the same parasites as *R. balthica* (Morley 2008; Karatayev et al. 2012). This might have led to a reduction of the parasite species diversity in BT with a reduced selection for the high MHC allele diversity perch had before enclosure. On the other hand, the enclosed perch population could have been subsequently confronted with new alien parasite species but lacking the compatible MHC alleles to defend them. Because of the isolated nature of the Biotest Lake perch population, there was no possibility to gain new alleles due to restricted migration of fish between populations and the time was too short for new mutations to change the set of effective MHC alleles. This scenario would result in a reduction of MHC allele diversity due to directional selection by parasites. Another possibility for the reduced number of MHC alleles would be a selective sweep scenario where one or a few parasite species were able to establish high densities followed by a frequency rise of one or a few MHC alleles in the host population, whereas other alleles got almost extinct. In addition, the higher temperature could be detrimental to some of the parasites directly causing their survival to decrease and hence pose less threat to the fish hosts. In general terms, this reinforces the conclusion that host–parasite interactions need to take parasite population dynamics into account as this can affect the dynamics and outcome of the host–parasite arms race substantially (Gokhale et al. 2013).

In conclusion, we have shown that the perch in the enclosed and heated Biotest Lake have experienced strong selection on the MHC class II receptor genes. We suggest, based on current data, that a shift in the parasite community as a result of the exceptionally high temperature in the Biotest Lake was accompanied by a shift in the MHC allele structure of the host population. However, even if this hypothesis seems to be the most likely given the data, the importance of other, unknown, ecological changes that have occurred as a result of the isolation cannot be excluded. We also found that the dynamics of MHC alleles over time can change in unpredictable ways in terms of induced cyclic behavior of one allele and reduced cyclic behavior of another allele. These results show that the biological effects of a change in environmental conditions are to a large extent unpredictable.

## Supporting Information

**Table S1.**MHC allele-frequencies in the Biotest (BT) and Forsmark (FM) between 1977 and 2009. **Table S2.** Number of alleles, expected heterozygosity (He), and Garza-Williamson M for all loci and years for the microsatellite data.

**Figure S1.** Saturation curve for sampling of MHC-alleles. **Data S1.** A test of the bias in the estimation of *F*_ST_ introduced by the unknown degree of heterozygosity.
