# Supplementary material for: Isolation over 35 years in a heated biotest basin causes selection on MHC class IIß genes in the European perch (Perca fluviatilis L.)
Source: Ecol Evol. 2015 Mar 5;5(7):1440–55. doi: 10.1002/ece3.1426 (PMC4395174; doi:10.1002/ece3.1426)
Supplement: Supplementary file 2 — Figure S1. Saturation curve for sampling of MHC-alleles. Data S1. A test of the bias in the estimation of FST introduced by the unknown degree of heterozygosity. [file ece30005-1440-sd2.docx]

S1. Saturation curve


Data S2. Test of the bias involved in calculating F_ST_.

We calculated the minimum and maximum number of possible loci for each individual based on the individual allele numbers found. The maximum number of loci in an individual was calculated as being equivalent to the number of different alleles found assuming that each allele is in the homozygous state. Likewise the minimum number of alleles was calculated assuming that all alleles are in the heterozygous state. Thus, if we found e.g. five alleles in an individual this could reflect either a maximum of five homozygous loci or a minimum of three loci (two heterozygous and one homozygous locus). Thus, in the “maximum number of loci” assumption we calculated allele frequencies with a maximum of ten alleles although we in reality only counted five alleles assuming that ten alleles are present in the individual due to the homozygous state of five loci present. Accordingly allele frequencies under the “minimum number of loci” assumption were calculated. Using both assumptions, we randomly drew the number of alleles from the range of possible alleles as described above and calculated allele frequencies and pairwise F_ST_-values. This was repeated 10 000 times to get 95 % intervals.

The open circles are the observed F_ST_-values, the black circles are the simulated F_ST_-values and the dotted lines the 95 % interval. The open squares represent the upper 97.5 % level based on sampling only. In short, the larger F_ST_-values are overestimated, while the smaller ones underestimated, but the magnitude of the bias is much lower than the uncertainty due to finite sample sizes.
